# Pharmacokinetics of Arctigenin and Fructus Arctii Powder in Piglets

**DOI:** 10.3389/fvets.2019.00235

**Published:** 2019-07-25

**Authors:** Bin He, Hai-Jing Zhang, Wen-Hai Yang, Zhi-Yong Shao, Li-Jun Wu, Xia-Bing Chen, Jie Chen, Wu Liu, Zhi-Ping Ran, Rr-Guang Jin, Ji-Yue Cao

**Affiliations:** ^1^Institute of Animal Husbandry and Veterinary, Wuhan Academy of Agricultural Sciences, Wuhan, China; ^2^Tianjin Baodi District Animal Husbandry and Aquaculture Development Service Center, Tianjin, China; ^3^College of Veterinary Medicine, Huazhong Agricultural University, Wuhan, China

**Keywords:** arctigenin, Fructus arctii powder, high-performance liquid chromatography, piglet, pharmacokinetics

## Abstract

Fructus arctii, also known as great power seed, is the dried fruit of *Arctium lappa* of the family Compositae. It is a commonly used veterinary herbal medicine, and arctigenin is the main active ingredient. The aim of this study was to characterize the absorption, distribution, metabolism, and excretion of arctigenin and Fructus arctii powder in piglets. These data were used to provide a theoretical reference for the development and clinical use of new veterinary drugs. Sixteen healthy piglets (mean weight 30.0 ± 5.0 kg) were divided into two groups. One group was administered 2.0 mg/kg body weight (bw) arctigenin intravenously, and the other was administered 1.0 g/kg^.^bw Fructus arctii powder by gavage. Blood samples were collected from the anterior vena cava at different time points, and the concentration of arctigenin in the plasma of the piglets was determined using high-performance liquid chromatography (HPLC). Arctigenin conformed to a two-compartment model with no absorption, and the main pharmacokinetic parameters were as follows: distribution half-life (*t*_1/2α_)−0.166 ± 0.022 h; elimination half-life (*t*_1/2β_)−3.161 ± 0.296 h; apparent volume of distribution (*V*_d_)−0.231 ± 0.033 L/kg; clearance rate (CL_b_)−0.057 ± 0.003 L/(h.kg); and area under the curve (AUC)−1.189 ± 0.057 g^.^h/mL. The pharmacokinetic parameters of arctigenin following oral administration of the Fructus arctii powder were as follows: absorption half-life (*t*_1/2ka_)−0.274 ± 0.102 h, *t*_1/2α_−1.435 ± 0.725 h, *t*_1/2β_−63.467 ± 29.115 h, *V*_d_−1.680 ± 0.402 L/kg, CL_b_−0.076 ± 0.028 L/(h kg), peak time (*t*_max_)−0.853 ± 0.211 h, peak concentration (*C*_max_)−0.430 ± 0.035 g/mL, and AUC−14.672 ± 4.813 g/mL. These results indicated that intravenous arctigenin was sparingly distributed in tissues. In contrast, orally administered Fructus arctii powder was rapidly absorbed, more widely distributed, and more slowly eliminated than the intravenous arctigenin, which may indicate its sustained pharmacological effects.

## Introduction

Fructus arctii, also known as great power seed, is the dried fruit of *Arctium lappa* of the family Compositae. It is a commonly used veterinary herbal medicine and is cold, pungent, and bitter in taste. It is associated with the lung and stomach meridians and can eliminate wind-heat and promote healing lung rashes. *Arctium lappa* is used by veterinarians to treat external wind-heat, cough, asthma, sore throat, and other conditions (1). The main active components of Fructus arctii are lignans, including arctiin and arctigenin (2). Arctiin can be converted, by gastrointestinal microorganisms, to arctigenin (3), which is the active component of Fructus arctii (4, 5). Arctigenin exerts anti-inflammatory (5, 6), antiviral (7, 8), antibacterial (9), antitumor (10, 11), and anti-diabetic (12) effects, and thus, it has excellent therapeutic potential. In this study, the pharmacokinetic characteristics of intravenously administered arctigenin and orally administered Fructus arctii powder in piglets were investigated to provide a theoretical basis and reference for the development and clinical use of arctigenin. Furthermore, this study will contribute to modernization of Chinese veterinary drugs.

## Materials and Methods

### Materials

#### Drugs

Arctigenin standard (98.1%; batch number, 160509) was purchased from Shanghai Ronghe Pharmaceutical Technology Co., Ltd. A standard solution containing 1.0 mg/mL arctigenin was prepared in a 5% methanol (aqueous) solution. An injection solution was prepared by dissolving arctigenin (10.0 mg/mL) in propylene glycol and water. Fructus arctii powder (0.33% arctigenin and 4.33% arctiin) was prepared by crushing Fructus arctii to 100 mesh granules. Arctiin and arctigenin content in Fructus arctii powder was determined per the methods in the veterinary pharmacopeia of the People's Republic of China. The dosage of arctigenin and Fructus arctii powder in piglets was determined according to the veterinary pharmacopeia of the People's Republic of China.

#### Instruments

The instruments used in this study were an Agilent 1200 high-performance liquid chromatography (HPLC) system equipped with an autosampler and ultraviolet detector (USA), a D3024R high-speed centrifuge (Scilogex, USA), an electronic balance (0.0001 g) (Sedoris, Germany), and an N-EVAP™ 111 Nitrogen Blowing Apparatus (Louis company, USA).

#### Animals

Sixteen healthy piglets (mean weight, 30.0 ± 5.0 kg, 60 days of age) were purchased from Wuhan Bomu Biotechnology Co., Ltd. They were randomly divided into two groups (nos. 1–8 and nos. 9–16). Each group consisted of four males (nos. 5–8 and 13–16) and four females (nos. 1–4 and 9–12). The piglets were housed in the animal experiment center of Wuhan Academy of Agricultural Sciences. The animal study was conducted per lab animal ethical review number 20160521031. The temperature of the pigsty was controlled at (24.0 ± 1.0°C), and the piglets were allowed free access to water and food. The feed was a diet without any drugs to prevent confounding results due to other drugs. Prior to execution of experiments, the piglets were fed normally for 2 weeks. After completion of the experiments, normal feeding was carried out.

### Method

#### Chromatographic Conditions

The chromatographic column, detection wavelength, mobile phase, flow rate, injection volume, and column temperature were Agilent SB-C_18_ (250 mm ×4.6 mm, 5 μm), 280 nm, methanol/acetonitrile/water (31:20:49 V/V/V), 0.8 ml/min, 30 μL, and 30 ± 0.1°C, respectively (13–15).

#### Plasma Sample Treatment

Plasma samples were stored in a −20°C freezer and were thawed at 20 ± 5°C. Samples (0.5 mL) were accurately transferred to 5.0-ml centrifuge tubes, 2.0 mL of dichloromethane was added, and the mixture was vortexed for 60 s. The mixture was centrifuged at 9,000 × *g* for 10.0 min to separate the dichloromethane. The dichloromethane layers were extracted twice using this method and combined. The dichloromethane extracts were placed in 15.0-mL centrifuge tubes and dried under nitrogen at 40°C in a water bath. The residue was dissolved in 200 μL of mobile phase, centrifuged at 9,000 × *g* for 10.0 min, and the supernatant was filtered through a 0.22-μm filter. The filtrate (30 μL) was injected onto the HPLC system for analysis (16–18).

#### Establishment of Plasma Standard Curve

Blank plasma (500 μL) was added to 5.0-mL polypropylene centrifuge tubes, and this was followed by a series of diluted arctigenin working standard solutions. The resulting spiked plasma samples contained 0.05, 0.1, 0.25, 0.5, 1.0, 2.5, and 5.0 μg/mL arctigenin. The samples were extracted using the procedure described in section Plasma Sample Treatment and analyzed using HPLC. The measured peak area (*x*) and concentration (*y*) of arctigenin were plotted, and the standard curve in plasma was established. The regression equation was used to calculate the correlation coefficient (*r*).

#### Method Verification

Blank plasma (500 μL) was transferred to 5.0-mL polypropylene centrifuge tubes. Diluted bovine aglycone working standard solutions at high, medium, or low concentrations were added, and the solutions were mixed well. The samples were prepared (5.0, 1.0, and 0.1 μg/mL) according to the section Plasma Sample Treatment and analyzed using HPLC. Five concentrations were analyzed each day to determine the intraday coefficient of variation. Repeat analyses were evaluated on five different days to determine the interday coefficient of variation. Furthermore, the recovery, accuracy, precision, and sensitivity of the method were calculated according to the following formulae:

Accuracy (extraction recovery) = peak area of drug injected after sample extraction/peak area after standard solution injection ×100%;

Precision (intraday and interday coefficient of variation) = standard deviation/average ×100%;

Limit of detection (LOD): The lowest concentration at which the signal-to-noise ratio (S/N) was ≥3;

Limit of quantification (LOQ): The minimum concentration at which S/N was ≥10, and the accuracy and precision were both within the acceptance criteria.

#### Pharmacokinetic Test Design

The pigs were fasted for 12.0 h prior to administration of arctigenin and Fructus arctii powder. Each piglet was weighed, and blood samples were collected from the anterior vena cava as blank controls. The first group of piglets (nos. 1–8) was administered 2.0 mg/kg^.^body weight (bw) arctigenin by intravenous injection, and the second group (nos. 9–16) was given 1.0 g/kg^.^bw Fructus arctii powder by gavage. In both groups, blood samples were collected from the anterior vena cava (5.0 mL) at 0.167, 0.333, 0.5, 0.75, 1.0, 1.5, 2.0, 3.0, 4.0, 5.0, 6.0, and 8.0 h for the first group, and at additional time points of 12.0, 24.0, and 48.0 h for the second group (nos. 9–16 piglets). Blood samples were collected in heparinized polypropylene centrifuge tubes. The samples were centrifuged for 15.0 min at 3,000 × *g*, and the supernatants were collected and stored at −20°C until pharmacokinetic analysis (19).

#### Determination of Arctigenin Concentration in Piglet Plasma

The plasma samples collected at each time point after arctigenin administration were analyzed by HPLC (according to the section Plasma Sample Treatment), the peak area of arctigenin was calculated, and the standard curve regression equation was used to calculate the plasma concentration of arctigenin.

#### Data Analysis

Excel software was used to generate the plasma standard curve and the concentration–time curve. Pharmacokinetic model fitting and pharmacokinetic parameters were analyzed using the 3p97 pharmacokinetic software created by a mathematical professional committee of the Chinese Pharmacological Society.

## Experimental Results

### Chromatographic Behavior of Arctigenin in Plasma

The retention time of arctigenin (ACT) was 14.0 min. The peak was symmetrical, the chromatogram showed a stable baseline, and the peak was well resolved from the impurity peak. The method also showed good sensitivity for arctigenin.

### Plasma Standard Curve

**Figure 1:**
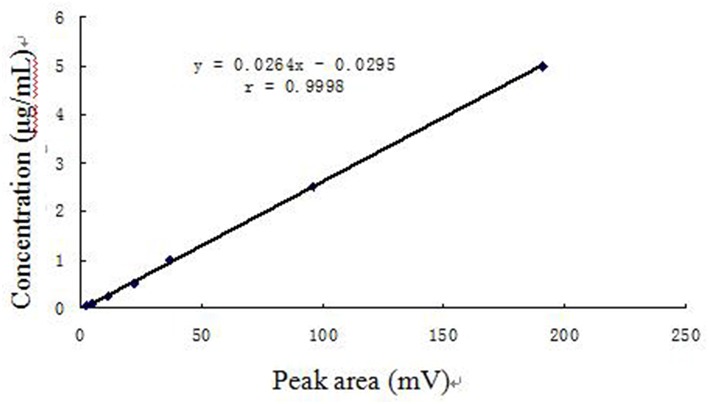
Calibration curve of arctigenin in piglet plasma.

The plasma concentration curve for arctigenin was linear (*r* = 0.9998) across the range of 0.05–10.0 μg/mL. The regression equation was *y* = 0.0265 *x* – 0.0328 (where *x* represents the peak area of arctigenin and *y* represents the plasma concentration of arctigenin). The calibration curve of arctigenin in piglet plasma is shown in Figure 1.

### Method Verification

The detection limit of arctigenin in piglet plasma was 0.025 μg/mL, and the lower LOQ was 0.05 μg/mL. The arctigenin response was linear across the range of 0.05–5.0 μg/mL, the regression equation was *y* = 0.0264 *x* – 0.0295, and the correlation coefficient was 0.9998. Recovery was 83.35% greater, the intraday variation was no more than 3.51%, and the interday variation was no more than 5.90%. Recovery, interday variation, and intraday variation are summarized in Tables 1, 2.

**Table 1:** Intraday recovery and precision values for the determination of arctigenin in piglet plasma (*n* = 5).

**Arctigenin concentration (μg/mL)**	**Recovery (%) ($\bar{x}$ ± S.D)**	**Coefficient of intraday variation (%)**
0.1	89.69 ± 2.79	3.11
1	91.19 ± 3.20	3.51
5	93.08 ± 1.98	2.13

**Table 2:** Interday recovery and precision values for the determination of arctigenin in piglet plasma (*n* = 5).

**Arctigenin concentration (μg/mL)**	**Recovery (%) ($\bar{x}$ ± S.D)**	**Coefficient of interday variation (%)**
0.1	83.35 ± 4.92	5.90
1	91.25 ± 4.36	4.77
5	88.99 ± 0.80	0.90

### Pharmacokinetic Test Data

#### Intravenous Arctigenin Injection

**Figure 2:**
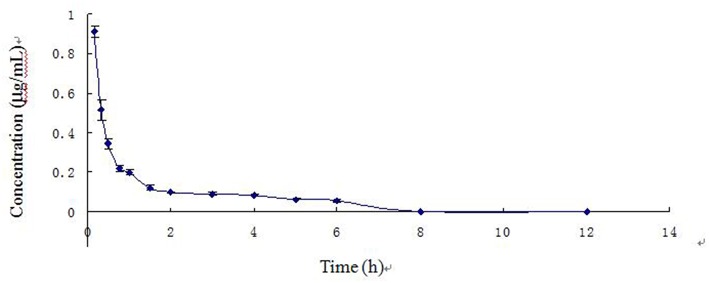
Blood concentration–time curve of arctigenin in piglets after intravenous injection (2.0 mg/kg).

The blood arctigenin concentration time course in piglets after a single intravenous injection of arctigenin (2.0 mg/kg.bw) is summarized in Table 3, the main pharmacokinetics parameters are summarized in Table 4, and the blood arctigenin concentration–time curve is shown in Figure 2.

**Table 3:** Blood concentration of arctigenin in piglets after intravenous injection (2.0 mg/kg.bw).

**Time (h)**	**Blood concentration per piglet (μg/mL)**								**Average blood concentration ($\bar{x}$ ± S.D.)**
	**1**	**2**	**3**	**4**	**5**	**6**	**7**	**8**	
0.167	0.913	0.924	0.942	0.874	0.934	0.891	0.937	0.876	0.911 ± 0.026
0.333	0.500	0.548	0.558	0.447	0.571	0.523	0.498	0.534	0.522 ± 0.037
0.5	0.338	0.309	0.359	0.367	0.321	0.364	0.346	0.371	0.347 ± 0.021
0.75	0.208	0.206	0.236	0.227	0.254	0.210	0.235	0.236	0.227 ± 0.016
1.0	0.200	0.182	0.211	0.203	0.193	0.181	0.186	0.179	0.192 ± 0.011
1.5	0.124	0.108	0.137	0.118	0.132	0.118	0.154	0.106	0.125 ± 0.015
2.0	0.097	0.081	0.110	0.092	0.087	0.094	0.106	0.086	0.094 ± 0.010
3.0	0.092	0.076	0.097	0.081	0.073	0.083	0.095	0.072	0.084 ± 0.010
4.0	0.081	0.071	0.086	0.076	0.068	0.073	0.081	0.064	0.075 ± 0.007
5.0	0.060	0.063	0.065	0.055	0.061	0.064	0.071	0.059	0.062 ± 0.004
6.0	0.054	0.052	0.060	0.053	0.051	0.055	0.063	0.051	0.055 ± 0.004
8.0	ND	ND	ND	ND	ND	ND	ND	ND	ND

*ND, not detected*.

**Table 4:** Pharmacokinetic parameters of arctigenin (2.0 mg/kg^.^bw) in piglets after intravenous injection.

**Pharmacokinetic parameter**	**Units**	**($\bar{x}$ ± S.D.)**
*A*	μg/mL	1.479 ± 0.191
α	h^−1^	4.194 ± 0.551
*B*	μg/mL	0.187 ± 0.023
β	h^−1^	0.221 ± 0.022
*V*_d_	L/kg	0.307 ± 0.033
*t*_1/2α_	h	0.166 ± 0.022
*t*_1/2β_	h	3.161 ± 0.296
K_21_	h^−1^	0.673 ± 0.085
K_10_	h^−1^	1.399 ± 0.163
K_12_	h^−1^	2.396 ± 0.349
AUC	μg^.^h/mL	1.189 ± 0.057
CL_b_	L/(h^.^kg)	0.068 ± 0.003

After intravenous injection of arctigenin, the plasma concentration–time data of the arctigenin piglets conformed to the non-absorbed two-compartment model with the formula *C* = 1.479e^−4.194t^ + 0.187e^−0.221t^. The distribution half-life (*t*_1/2α_) was 0.166 ± 0.022 h, the elimination half-life (*t*_1/2β_) was 3.161 ± 0.296 h, and the apparent volume of distribution (*V*_d_) was 0.231 ± 0.033 L/kg. These results suggested that, in piglets, burdock glycosides were distributed rapidly, but distribution into tissues was low and elimination was rapid due to metabolism.

#### Oral Administration (by Gavage) of Fructus Arctii Powder

**Figure 3:**
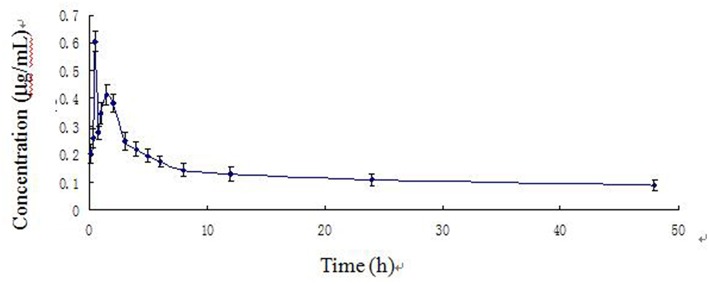
Arctigenin concentration–time curve in piglets after a single oral dose of Fructus arctii powder (1.0 g/kg^.^bw).

The time course of blood arctigenin concentration in piglets fed a single dose of Fructus arctii powder (1.0 g/kg^.^bw) is shown in Table 5, the main pharmacokinetic parameters are shown in Table 6, and the blood arctigenin concentration–time curve is shown in Figure 3.

**Table 5:** Blood concentration (μg/mL) of arctigenin in piglets after a single oral dose of Fructus arctii powder (1.0 g/kg.bw).

**Time (h)**	**Blood concentration per piglet (μg/mL)**								**Average blood concentration ($\bar{x}$ ± S.D.)**
	**9**	**10**	**11**	**12**	**13**	**14**	**15**	**16**	
0.167	0.179	0.187	0.161	0.216	0.242	0.195	0.166	0.250	0.200 ± 0.031
0.333	0.258	0.271	0.242	0.274	0.306	0.216	0.211	0.282	0.258 ± 0.031
0.5	0.594	0.572	0.633	0.665	0.625	0.549	0.609	0.601	0.606 ± 0.034
0.75	0.279	0.250	0.295	0.240	0.285	0.322	0.290	0.256	0.277 ± 0.025
1.0	0.401	0.285	0.359	0.335	0.306	0.369	0.335	0.390	0.348 ± 0.037
1.5	0.454	0.385	0.382	0.398	0.364	0.422	0.459	0.440	0.413 ± 0.034
2.0	0.433	0.369	0.337	0.385	0.343	0.401	0.396	0.417	0.385 ± 0.032
3.0	0.213	0.242	0.258	0.205	0.242	0.279	0.301	0.237	0.247 ± 0.030
4.0	0.195	0.211	0.240	0.192	0.229	0.258	0.200	0.227	0.219 ± 0.022
5.0	0.176	0.187	0.216	0.179	0.163	0.229	0.195	0.203	0.194 ± 0.020
6.0	0.147	0.169	0.203	0.158	0.161	0.198	0.161	0.192	0.174 ± 0.020
8.0	0.145	0.139	0.126	0.134	0.108	0.187	0.158	0.153	0.144 ± 0.022
12.0	0.132	0.121	0.124	0.100	0.103	0.184	0.142	0.132	0.130 ± 0.025
24.0	0.105	0.095	0.100	0.089	0.079	0.142	0.124	0.126	0.108 ± 0.020
48.0	0.100	0.071	0.095	0.066	0.077	0.121	0.079	0.105	0.089 ± 0.018

**Table 6:** The main pharmacokinetic parameters in piglets after a single oral dose of Fructus arctii powder (1.0 g/kg^.^bw).

**Pharmacokinetic parameter**	**Unit**	**($\bar{x}$ ± S.D.)**
*A*	μg/mL	0.644 ± 0.242
α	h^−1^	0.567 ± 0.213
*B*	μg/mL	0.160 ± 0.036
β	h^−1^	0.013 ± 0.005
*k*_α_	h^−1^	3.400 ± 2.881
*V*_d_	L/kg	1.680 ± 0.402
*t*_1/2α_	h	1.435 ± 0.725
*t*_1/2β_	h	63.467 ± 29.115
*t*_1/2ka_	h	0.274 ± 0.102
K_21_	h^−1^	0.155 ± 0.060
K_10_	h^−1^	0.047 ± 0.023
K_12_	h^−1^	0.378 ± 0.149
AUC	μg^.^h/mL	14.672 ± 4.813
CL_b_	L/(h^.^kg)	0.076 ± 0.028
*t*_max_	h	0.853 ± 0.211
*C*_max_	μg/mL	0.430 ± 0.035

The blood arctigenin concentration–time data adhered to the absorption chamber model. The formula was *C* = 0.644e^−0.567t^ + 0.160e^−0.013t^ – 0.864e^−1.826t^, the curve was bimodal, *t*_max_ was 0.853 ± 0.211 h, the maximum concentration (*C*_max_) was 0.430 ± 0.035 μg/mL, the absorption half-life (*t*_1/2ka_) was 0.274 ± 0.102 h, the elimination half-life (*t*_1/2β_) was 63.467 ± 29.115 h, and the apparent volume of distribution (*V*_d_) was 1.680 ± 0.402 L/kg. The powder was rapidly absorbed, widely distributed, and was slowly eliminated.

## Discussion

Arctigenin has excellent therapeutic potential. This study evaluated the pharmacokinetics of arctigenin and Fructus arctii to characterize their absorption, distribution, metabolism, and excretion in piglets and provides a theoretical basis and reference for the development and clinical use of new veterinary drugs.

After intravenous injection of arctigenin, the distribution half-life (*t*_1/2α_) (0.166 ± 0.022 h) was short and the apparent distribution volume (*V*_d_) (0.307 ± 0.033 L/kg) was low, indicating that arctigenin was rapidly distributed, but distribution into tissues was relatively low. Arctigenin was mainly distributed in the blood and the extracellular fluid (19). The elimination half-life (*t*_1/2β_) (3.161 ± 0.296 h) was relatively short, indicating that elimination occurred rapidly and did not correlate with distribution.

The absorption half-life (*t*_1/2ka_) of Fructus arctii powder was 0.274 ± 0.102 h and the peak time (*t*_max_) was 0.853 ± 0.211 h, indicating that absorption was rapid and the time to reach the maximum blood concentration was short. The *t*_1/2β_ (63.467 ± 29.115 h) was long, the *V*_d_(1.680 ± 0.402 L/k) was large, and the peak concentration *C*_max_ (0.430 ± 0.035 μg/mL) was relatively low. These results indicate that arctigenin was eliminated slowly and was distributed to tissues, resulting in a relatively low concentration of the drug in the blood, which may indicate a sustained pharmacological effect (17). A previous report showed that arctigenin was rapidly absorbed in rats and beagle dogs (absorption rate < 1 h), showed a high degree of absorption (absolute bioavailability > 100%), and was rapidly eliminated. The time course of tissue distribution of arctigenin in rats after intravenous administration was indicative of rapid (2.5 h to reach the peak concentration) and wide (detectable in almost all tissues and organs) distribution. The arctigenin concentration was highest in the intestine, followed by that in the heart, liver, pancreas, and kidney (20). This was similar to the rapid and wide distribution we observed following oral administration of Fructus arctii to piglets. Comparison of pharmacokinetic parameters of arctigenin and Fructus arctii, it was found that the main differences in pharmacokinetic parameters were elimination half-life (*t*_1/2β_), apparent volume of distribution (*V*_d_), and area under the concentration–time curve (AUC). These differences were significant (*p* < 0.01). These results indicated that the gastrointestinal system greatly influenced the absorption, distribution, metabolism, and excretion of arctigenin.

After intravenous injection of arctigenin, the plasma concentration–time data in piglets conformed to the non-absorbed two-compartment model. These results were similar to those of a previous study, which evaluated intravenous injection of different doses of arctigenin and its valine ester derivative in Wistar rats (21). The blood drug concentration–time curve was consistent with the two-compartment model. After oral administration of Fructus arctii, the blood concentration–time data of arctigenin in piglets were consistent with the two-compartment model of absorption. This is not in line with the characteristics of the one-compartment model after Wistar rats were orally administered different doses of arctiin and its valine ester derivative, which was reported by Cai et al. (21). These differences may have been species specific. The blood arctigenin concentration–time curve of Fructus arctii powder was bimodal. The peak times (*T*_max_) of the first and second curves were 0.5 and 1.5 h, at which the peak concentrations (*C*_max_) were 0.606 ± 0.036 and 0.413 ± 0.036 μg/ml, respectively. This bimodal phenomenon was also observed in a pharmacodynamic study of Fructus arctii in mice by Yuan (22). Additionally, the author found that there were two peak concentrations, which indicated that the absorption process of the drug was unique and that the pharmacodynamics were related to the pharmacokinetics. The authors suggested that the two peaks might have resulted from the conversion of arctiin in the aqueous extract to arctigenin or the enterohepatic circulation of arctigenin. Gao et al. reported that arctigenin undergoes extensive glucuronic acid hydrolysis in the liver, intestine, and plasma, which may be associated with the dual absorption peaks of the drug, further suggesting that the drug remains in the body for a prolonged period (23). Ikeda et al. reported that the pharmacokinetics of arctigenin in the human body followed a non-linear model. This study observed a second absorption peak for arctiin and arctigenin, suggesting intrahepatic circulation (24). Gao et al. showed that glucaldehyde acidification is the main intestinal and liver metabolic pathway for arctigenin, and arctigenin excreted in the bile can be further hydrolyzed to arctiin, indicating potential enterohepatic circulation (25). Gao et al. found that hydrolysis was the main metabolic pathway of arctigenin; subsequently, arctigenic acid, arctigenin 4-*O*-glucuronide, and 4-*O*-demethylarctigenin were identified as three novel metabolites (26). These reports were similar to those indicating double absorption peaks of arctigenin in piglets following oral administration of Fructus arctii, which provided further support for hepatic and intestinal circulation of arctigenin in piglets.

Zeng et al. found that the function of p-glycoprotein was impaired in diabetic rats and that arctigenin is a substrate for p-glycoprotein, resulting in increases of 356.8 and 223.4% in the *C*_max_ and AUC_0−10h_ values of arctigenin administered orally to diabetic rats compared with normal rats, respectively. However, after intravenous injection, there were no significant differences in *C*_max_ or AUC_0−10h_ between normal and diabetic rats (27). The blood drug concentrations observed in the previous experiment by Zeng et al. and the present study were not affected by the physiological state of the piglets or the gender of the piglets. Furthermore, no significant differences were observed in the pharmacokinetic parameters between the two studies; presumably, the pharmacology and pharmacokinetics of arctigenin after oral or intravenous administration are not affected by physiological conditions, whether pathological or normal. However, the reality needs further investigation.

Pharmacokinetic studies of traditional Chinese medicines often result in bimodal absorption time courses due to the complex nature of these medicines and conversion of components to the same parent molecule (28). In a study by Guo et al. on the pharmacokinetics of orally administered baicalin and Radix Scutellariae in rats, the blood drug concentration–time curve was bimodal. This may have been due to the hydrolysis of baicalin to its glycoside form by intestinal flora, resulting in enterohepatic circulation (29). Furthermore, this phenomenon may be associated with mother nuclear components sharing metabolic mechanisms (30). Yang et al. studied the pharmacokinetic characteristics of cinnamic acid in baoxin pellets in rats and found that the blood drug concentration–time curve was bimodal. Cinnamic aldehyde can be oxidized to cinnamic acid *in vivo*, and reabsorption resulted in a second absorption peak, which may have been indicative of enterohepatic circulation (31). The bimodal phenomenon observed in this study was likely caused by metabolism of arctiin to arctigenin. It is possible that the second peak resulted from enterohepatic circulation or through conversion of arctiin to arctigenin. The specific mechanism responsible for the bimodal absorption distribution of arctigenin should be investigated in future studies.

## Conclusion

In this study, HPLC was used to quantitate arctigenin in piglet plasma. The method was sensitive, specific, and rapid. This method can be used to support preclinical pharmacokinetic studies of arctigenin with new veterinary drugs. The pharmacokinetic characteristics of Fructus arctii and its main active ingredient arctigenin in piglets were characterized. The results showed that intravenous administration of arctigenin exhibited a two-compartment model without absorption in piglets and showed poor tissue distribution, short half-life, and rapid elimination. Oral administration of Fructus arctii exhibited a two-compartment model of arctigenin absorption characterized by fast absorption, wide distribution, and slow elimination, with potential hepatoenteric circulation, which suggested that arctigenin may exert effects for a prolonged period of time. Dosing amount and frequency should be controlled to prevent side effects caused by drug accumulation in the body due to slow elimination.

## Data Availability

All datasets generated for this study are included in the manuscript and/or the supplementary files.

## Ethics Statement

There is no violation of ethics or animal welfare in this study. The experiment is authorized by Wuhan academy of agricultural sciences and is willing to accept social supervision.

## Author Contributions

All authors listed have made a substantial, direct and intellectual contribution to the work, and approved it for publication.

### Conflict of Interest Statement

The authors declare that the research was conducted in the absence of any commercial or financial relationships that could be construed as a potential conflict of interest.
